# Imaging of reactive oxygen species using [^3^H]hydromethidine in mice with cisplatin-induced nephrotoxicity

**DOI:** 10.1186/s13550-015-0116-0

**Published:** 2015-07-11

**Authors:** Nozomi Takai, Kohji Abe, Misato Tonomura, Natsumi Imamoto, Kazumi Fukumoto, Miwa Ito, Sotaro Momosaki, Kae Fujisawa, Kenji Morimoto, Nobuo Takasu, Osamu Inoue

**Affiliations:** Department of Drug Metabolism & Pharmacokinetics, Research Laboratory for Development, Shionogi & Co., Ltd., 3-1-1 Futaba-cho, Toyonaka Osaka, 561-0825 Japan; Department of Applied Chemistry & Analysis, Research Laboratory for Development, Shionogi & Co., Ltd., 3-1-1 Futaba-cho, Toyonaka Osaka, 561-0825 Japan; Department of Drug Safety Evaluation, Research Laboratory for Development, Shionogi & Co., Ltd., 3-1-1 Futaba-cho, Toyonaka Osaka, 561-0825 Japan; Hanwa Intelligent Medical Center, Hanwa Daini Senboku Hospital, 3176 Fukaikita-machi, Naka-ku, Sakai, Osaka 599-8271 Japan; Division of Health Sciences, Osaka University Graduate School of Medicine, 1-7 Yamadaoka, Suita, Osaka 565-0871 Japan

**Keywords:** Cisplatin, Hydroxyl radical, Nephrotoxicity, Reactive oxygen species

## Abstract

**Background:**

Reactive oxygen species (ROS) have been implicated in cisplatin-induced nephrotoxicity. The aim of this study was to investigate the potential of using [^3^H]-labeled *N*-methyl-2,3-diamino-6-phenyl-dihydrophenanthridine ([^3^H]hydromethidine) for ex vivo imaging of regional ROS overproduction in mouse kidney induced by cisplatin.

**Methods:**

Male C57BL/6 J mice were intraperitoneally administered with a single dose of cisplatin (30 mg/kg). Renal function was assessed by measuring serum creatinine and blood urea nitrogen (BUN) levels and morphology by histological examination. Renal malondialdehyde levels were measured as a lipid peroxidation marker. Autoradiographic studies were performed with kidney sections from mice at 60 min after [^3^H]hydromethidine injection.

**Results:**

Radioactivity accumulation after [^3^H]hydromethidine injection was observed in the renal corticomedullary area of cisplatin-treated mice and was attenuated by pretreatment with dimethylthiourea (DMTU), a hydroxyl radical scavenger. Cisplatin administration significantly elevated serum creatinine and BUN levels, caused renal tissue damage, and promoted renal lipid peroxidation. These changes were significantly suppressed by DMTU pretreatment.

**Conclusions:**

The present study showed that [^3^H]hydromethidine was rapidly distributed to the kidney after its injection and trapped there in the presence of ROS such as hydroxyl radicals, suggesting that [^3^H]hydromethidine is useful for assessment of the renal ROS amount in cisplatin-induced nephrotoxicity.

## Background

Overproduction of reactive oxygen species (ROS) plays a crucial role in modulation of signal transduction and is involved in the pathogenesis of various diseases [1]. ROS have been implicated in drug-induced nephrotoxicity [2–5]. Several drugs induced lipid peroxidation in the kidney, and antioxidant nutrients such as vitamin E suppressed the renal damage. One electron reduction of molecular oxygen yields the superoxide anion (O_2_^−^), followed by production of hydrogen peroxide (H_2_O_2_) through spontaneous or superoxide dismutase-catalyzed dismutation. O_2_^−^ reacts with ferric ion to produce ferrous ion, which subsequently yields the hydroxyl radical (^·^OH) via Fenton reaction with H_2_O_2_. ^·^OH is known as the most reactive ROS, which causes DNA damage and lipid peroxidation [6, 7].

Cisplatin (*cis*-diamminedichloroplatinum [II]) is a widely used chemotherapeutic agent for cancers, but its clinical use is often limited due to its nephrotoxicity including acute kidney injury [8]. The mechanisms underlying cisplatin-induced nephrotoxicity are not completely understood, but previous works with dimethylthiourea (DMTU), a hydroxyl radical scavenger, have shown its protective effect against renal dysfunction and tissue damage in cisplatin-treated mice or rats, suggesting the involvement of ^·^OH in cisplatin-induced nephrotoxicity [9–12]. For further investigation of the relationship between ROS production and the pathogenesis of cisplatin-induced nephrotoxicity, a highly sensitive molecular probe for ROS detection in vivo would be a useful tool.

Techniques based on chemiluminescence [13–15] or fluorescence [16–21] from oxidizable molecular probes have been widely used for ROS detection in biological tissue samples or living animals. These optical imaging techniques employ a simple method with selectivity for a certain biological reaction, but there is a limitation when considering human studies and quantitative analysis due to signal attenuation with tissue depth.

Recently, we have reported on a novel probe, [^3^H]-labeled *N*-methyl-2,3-diamino-6-phenyl-dihydrophenanthridine ([^3^H]hydromethidine), as a radical trapping radiotracer [22]. [^3^H]hydromethidine is a derivative of hydroethidine, one of the most commonly used fluorescent probes for ROS detection [18–21]. We previously investigated the capability of [^3^H]hydromethidine for detection of ROS generated by sodium nitroprusside (SNP) microinjection into the striatum of mice, and the findings suggested that [^3^H]hydromethidine was rapidly distributed to the brain as well as peripheral organs, converted to its oxidized form through reaction with the ROS generated, and trapped in the tissue, while unreacted [^3^H]hydromethidine was immediately eliminated from normal tissue. Thus, [^3^H]hydromethidine has a preferable profile for in vivo ROS detection in a variety of pathological conditions, and ^11^C-labeling instead of ^3^H-labeling could allow ROS imaging in living animals or humans with positron emission tomography (PET). Here we report the use of [^3^H]hydromethidine for ROS detection in cisplatin-induced nephrotoxicity and the involvement of ^·^OH under DMTU treatment.

## Methods

### Animals

All animal experiments in the present study were reviewed and approved by the Institutional Animal Care and Use Committee of Shionogi Research Laboratories (Osaka, Japan). Male C57BL/6 J mice, weighing 18.2–24.6 g, were purchased from CLEA Japan, Inc. (Tokyo, Japan), housed in a temperature-controlled room maintained on a 12-h light/dark cycle with lights on at 8:00 am, and allowed free access to chow and tap water. At 8 weeks of age, mice of the cisplatin group were intraperitoneally administered with a single dose of cisplatin (30-mg/kg body weight, Wako Pure Chemical Industries Ltd., Osaka, Japan) dissolved in saline at 3 mg/mL. This dose of cisplatin was selected based on previous studies using mice with cisplatin-induced nephrotoxicity [10, 23]. Mice of the cisplatin + DMTU group were intraperitoneally administered with DMTU (100-mg/kg body weight, Sigma-Aldrich, St. Louis, MO, USA) dissolved in saline at 10 mg/mL, 30 min prior to the cisplatin administration (30 mg/kg). The control + DMTU mice were administered with DMTU (100 mg/kg) alone. For the histopathological examination, DMTU was injected into mice 30 min prior to the cisplatin administration and then injected once a day until 72 h after cisplatin administration, as described in a previous report [10]. The groups of mice at 5, 10, or 18 h after cisplatin administration were used for autoradiographic study (*n* = 4–6). The groups of mice at 24 h after cisplatin administration were used for assessment of renal function and lipid peroxidation (*n* = 6). The groups of mice at 72 h after cisplatin administration were used for histopathological examination (*n* = 6–10).

### Synthesis of [^3^H]hydromethidine

[^3^H]hydromethidine (specific activity; 74 GBq/mmol, radiochemical purity; 98.2 %) was synthesized by *N*-methylation using [methyl-^3^H]Methyl nosylate as described previously [22]. [^3^H]hydromethidine was diluted with distilled water containing 5 % DMSO (v/v), giving 97.8 % of radiochemical purity.

### Autoradiographic study with kidney sections from mice injected with [^3^H]hydromethidine

An aqueous solution (5 % DMSO, v/v) of [^3^H]hydromethidine (185 kBq) was injected intravenously into the tail vein of mice at 5, 10, or 18 h after cisplatin administration. The mice were sacrificed by decapitation at post-injection time points of 1 or 60 min under deep anesthesia with isoflurane. The kidney was rapidly removed and frozen, and sections (20-μm thick) were prepared using a cryostat. The sections were exposed to an imaging plate (BAS-TR, GE Healthcare, Buckinghamshire, UK) for 14 days. After exposure, the plates were read with a FLA-3000 (Fujifilm Corp., Tokyo, Japan). Regions of interest (ROIs) were drawn on the corticomedullary junction [24], and the photo-stimulated luminescence value for each ROI (PSL/mm^2^) was determined using Multi Gauge version 2.3 (Fujifilm Corp.). The radioactivity concentrations in each ROI were expressed as (PSL − background)/area (mm^2^) [(PSL − BG)/mm^2^].

### Sample collection for assessment of renal function, lipid peroxidation, and histopathology

Apart from the autoradiographic study, mice from each group were euthanized by isoflurane anesthesia and exsanguinated via the inferior vena cava at 24 or 72 h after cisplatin administration. Blood samples were collected for assessment of renal function. Kidneys were stored at −80 °C until measurement of lipid peroxidation or fixed with 10 % neutral buffered formalin for histopathological examination.

### Assessment of renal function

The blood samples were centrifuged at 1000×*g* for 20 min at 4 °C to obtain serum. Blood urea nitrogen (BUN) and creatinine levels in the serum were measured using the urea nitrogen B-test kit (Wako Pure Chemical Industries Ltd.) and creatinine colorimetric/fluorometric assay kit (BioVision, Inc., Milpitas, CA, USA), respectively.

### Histopathological examination

The formalin-fixed kidney samples were embedded in paraffin. Paraffin sections were stained with hematoxylin and eosin using standard methodologies. The tissues were examined under a light microscope.

### Measurement of lipid peroxidation

The kidney samples were homogenized in ice-cold phosphate buffered saline (0.01 M, pH 7.4) containing 1-mM ethylenediaminetetraacetic acid to obtain 10 % (w/v) homogenate. The homogenate was centrifuged at 10,000×*g* for 5 min at 4 °C, and the supernatant was used for the determination of the malondialdehyde (MDA) level, an index of lipid peroxidation, using an MDA assay kit based on reaction with thiobarbituric acid (Northwest Life Science Specialties LLC, Vancouver, WA, USA).

### Statistical analysis

Data were expressed as mean ± SD. Statistical differences between the two groups were determined by an unpaired *t*-test. *P* < 0.05 was considered statistically significant.

## Results

### Renal distribution of radioactivity after intravenous injection of [^3^H]hydromethidine in cisplatin-treated mice

**Fig. 1:**
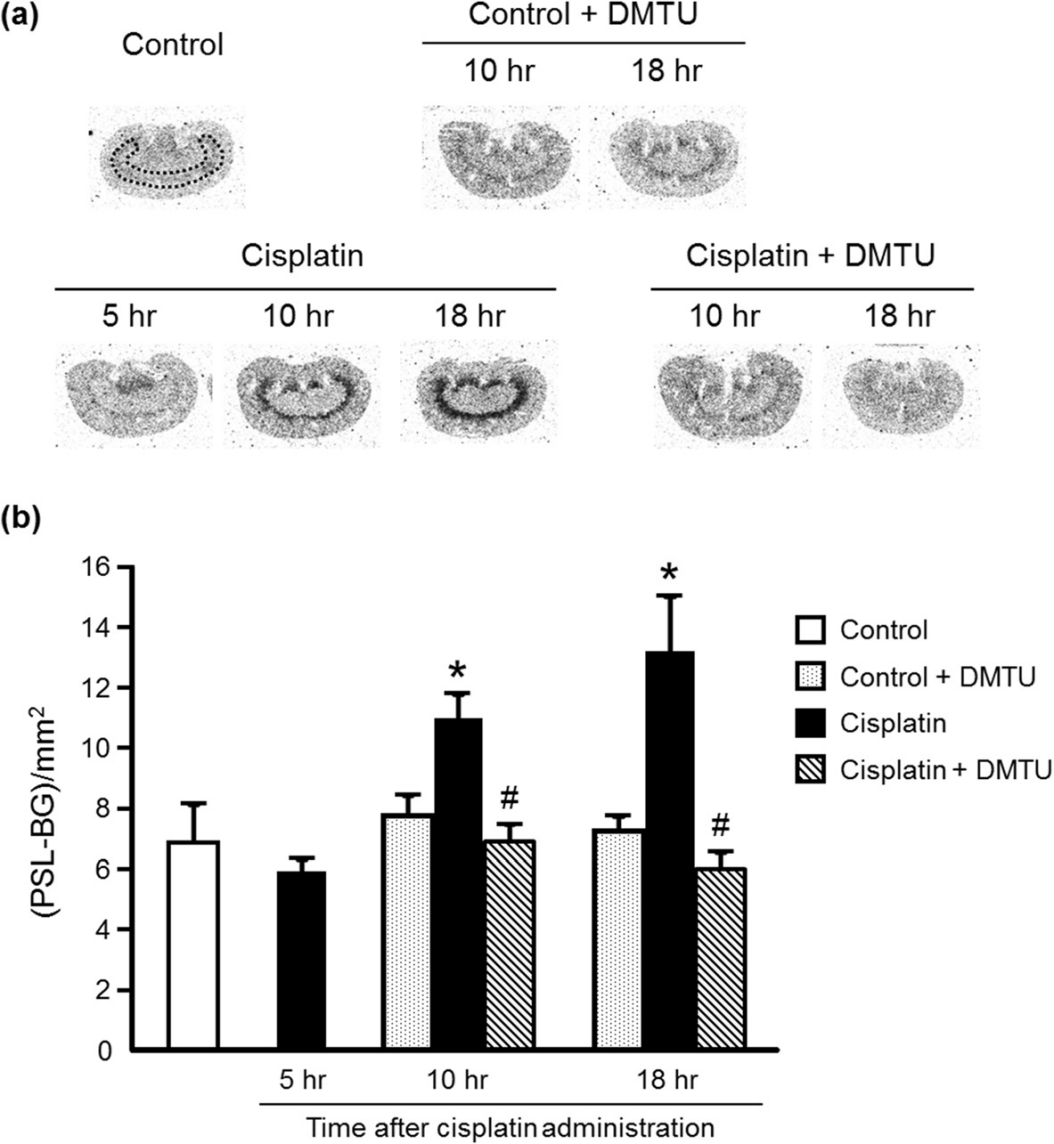
Radioactivity distribution in the kidney obtained at 60 min after [^3^H]hydromethidine injection. Typical autoradiograms of kidney sections from mice at 5, 10, or 18 h after cisplatin administration (**a**), and the results of quantitative analysis (**b**) are shown. ROIs were drawn on the corticomedullary junction as illustrated with a *dotted line* in the autoradiogram of control kidney. Data are expressed as mean ± SD (*n* = 4–6). *Significantly different from the control group (*P* < 0.05); # significantly different from the cisplatin group for each observed period (*P* < 0.05)

Figure 1 shows typical autoradiograms of kidneys obtained at 60 min after [^3^H]hydromethidine injection to mice. There was no difference in radioactivity accumulation between the control (6.85 ± 1.29 (PSL − BG)/mm^2^) and the cisplatin groups until 5 h after cisplatin administration (5.92 ± 0.42 (PSL − BG)/mm^2^). However, significantly high accumulation of radioactivity was observed in the corticomedullary junction of the kidney obtained from the cisplatin group at 10 or 18 h after cisplatin administration compared with the control group (10 h; 11.0 ± 0.8 (PSL − BG)/mm^2^, 18 h; 13.2 ± 1.8 (PSL − BG)/mm^2^). The radioactivity accumulation in the corticomedullary junction was significantly attenuated by pretreatment with DMTU (10 h; 6.88 ± 0.59 (PSL − BG)/mm^2^, 18 h; 5.97 ± 0.58 (PSL − BG)/mm^2^). The DMTU treatment alone did not alter renal radioactivity accumulation in the control + DMTU group (10 h; 7.78 ± 0.66 (PSL − BG)/mm^2^, 18 h; 7.25 ± 0.50 (PSL − BG)/mm^2^).

**Fig. 2:**
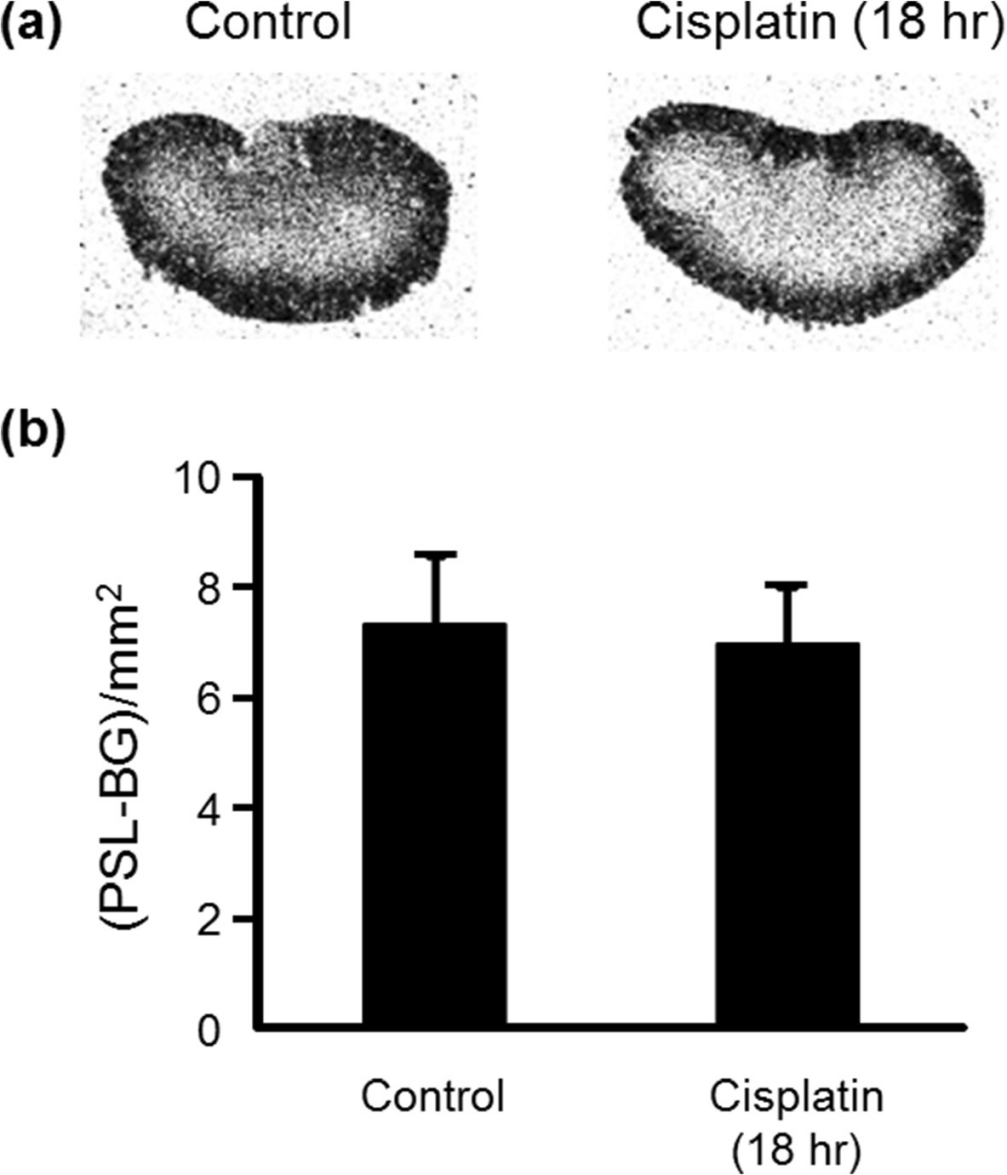
Radioactivity distribution in the kidney obtained at 1 min after [^3^H]hydromethidine injection. Typical autoradiograms of kidney sections from mice at 18 h after cisplatin administration, (**a**) and the results of quantitative analysis (**b**) are shown. ROIs were drawn on the whole tissue area. Data are expressed as mean ± SD (*n* = 4). No significant difference was observed between the two groups (*P* = 0.68)

Typical autoradiograms of kidneys obtained at 1 min after [^3^H]hydromethidine injection to mice in the control or the cisplatin group (18 h after cisplatin administration) are shown in Fig. 2. A very high accumulation of radioactivity in the cortical area was observed. There was no marked difference in radioactivity distribution between the two groups.

### Renal function and histopathology in cisplatin-treated mice

**Fig. 3:**
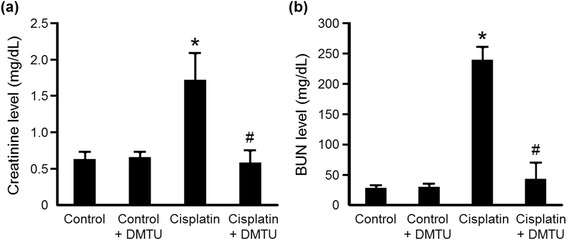
Renal function in cisplatin-treated mice. Serum creatinine (**a**) and BUN (**b**) levels were measured at 24 h after cisplatin administration. Data are expressed as mean ± SD (*n* = 6). *Significantly different from the control group (*P* < 0.05); # significantly different from the cisplatin group (*P* < 0.05)

**Fig. 4:**
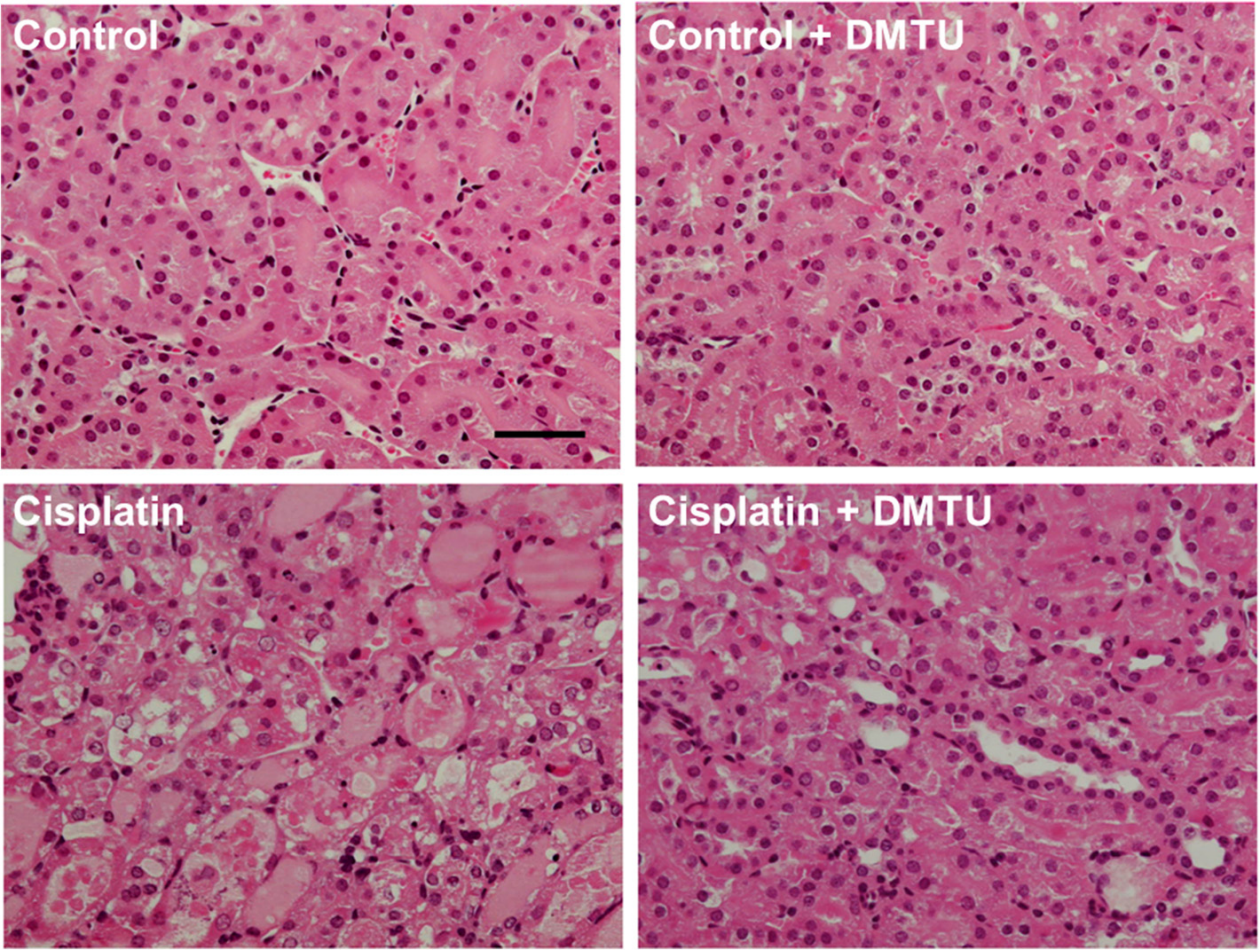
Representative micrographs of renal histopathology in mice at 72 h after cisplatin administration. Tubular necrosis, dilatation, and hyaline cast were observed in the cisplatin group, and the DMTU treatment suppressed the severity of the tissue damage. *Bar* = 50 μm

As shown in Fig. 3, BUN and creatinine levels in the serum obtained at 24 h after cisplatin administration were significantly elevated in the cisplatin group (BUN; 240 ± 20 mg/dL, creatinine; 1.72 ± 0.37 mg/dL) compared with the control group (BUN; 27.9 ± 4.3 mg/dL, creatinine; 0.630 ± 0.097 mg/dL), while there was no difference between the control and the control + DMTU groups (BUN; 29.8 ± 5.2 mg/dL, creatinine; 0.657 ± 0.072 mg/dL). The cisplatin-induced renal failure was significantly attenuated by pretreatment with DMTU (BUN; 43.0 ± 26.6 mg/dL, creatinine; 0.584 ± 0.168 mg/dL). The cisplatin-induced nephrotoxicity and the protective effect of DMTU were investigated by histopathological examination of kidneys obtained at 72 h after cisplatin administration because no obvious tissue damage was observed from the histopathological data at 24 h after cisplatin administration in our preliminary study. As shown in Fig. 4, cisplatin administration resulted in renal tissue damage including tubular necrosis, dilatation, and hyaline cast, whereas the control and the control + DMTU mice showed normal renal morphology. The severity of renal injury was suppressed by the treatment with DMTU.

### Renal lipid peroxidation in cisplatin-treated mice

**Fig. 5:**
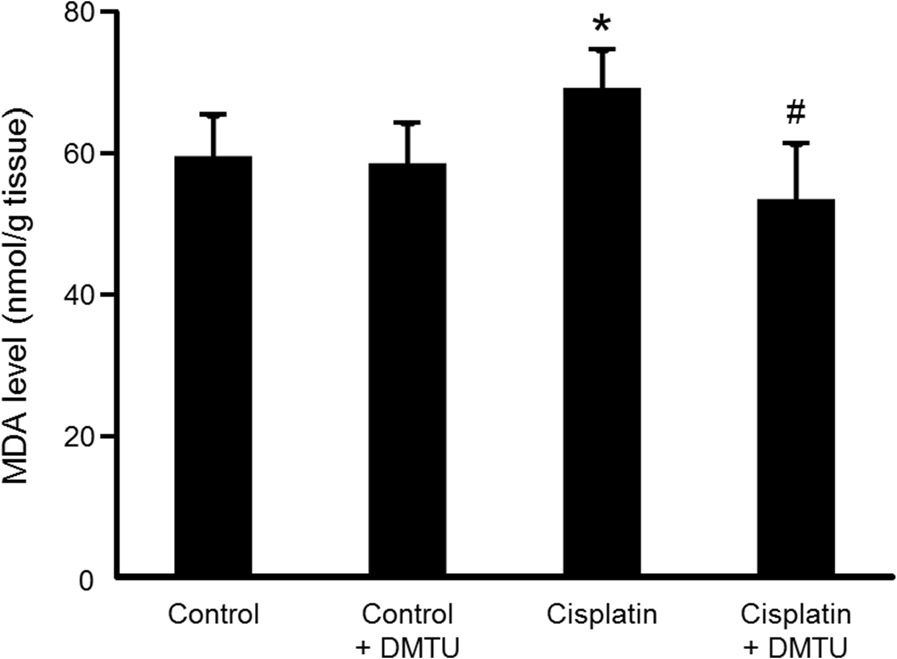
Renal lipid peroxidation in cisplatin-treated mice. MDA levels were measured at 24 h after cisplatin administration. Data are expressed as mean ± SD (*n* = 6). *Significantly different from the control group (*P* < 0.05); # significantly different from the cisplatin group (*P* < 0.05)

As shown in Fig. 5, MDA levels in the kidney obtained at 24 h after cisplatin administration significantly increased in the cisplatin group (69.1 ± 5.4 nmol/g tissue) compared with the control group (59.5 ± 5.9 nmol/g tissue), while there was no difference between the control and the control + DMTU groups (58.6 ± 5.6 nmol/g tissue). The DMTU pretreatment significantly reduced the cisplatin-induced increase of MDA levels (53.5 ± 7.8 nmol/g tissue).

## Discussion

Cisplatin is an effective chemotherapeutic agent for the treatment of solid tumors. However, its nephrotoxicity is a common side effect. In the present study, a single intraperitoneal administration of cisplatin at 30 mg/kg to mice increased serum creatinine and BUN levels and affected renal morphology, which was consistent with previous reports including clinical studies [25–27]. Cisplatin is eliminated via the kidney by glomerular filtration and tubular secretion, and the concentration in proximal tubular epithelial cells is much higher than the serum concentration, most likely due to the involvement of the copper transporter Ctr1 and the organic cation transporter OCT2 expressed in the kidney [28, 29]. The intracellularly accumulated cisplatin leads to nuclear and mitochondrial DNA damage and ROS production, followed by activation of necrotic and apoptotic pathways [8]. ROS causes cell injury through the peroxidation of lipid components of the cell membrane. Although previous studies suggest a relationship between cisplatin-induced nephrotoxicity and excess production of ROS such as ^·^OH, the exact roles of ROS in the kidney have not been understood [9–12]. Radioisotopic techniques such as PET are possible methods for studying the physiological roles of ROS in various states including nephrotoxicity. Recently, Chu et al. reported an ^18^ F-labeled derivative of hydroethidine for selective detection of O_2_^−^ in vivo [30]. Carroll et al. reported a boronate-caged [^18^ F]fluorothymidine for H_2_O_2_ detection [31]. We also reported a radical trapping radiotracer, [^3^H]hydromethidine, for ROS detection in biological tissues [22]. The results of in vitro studies showed that [^3^H]hydromethidine reacted with O_2_^−^ and ^·^OH, followed by conversion to its highly polar form. [^3^H]hydromethidine was rapidly distributed to organs such as the brain, heart, lung, and kidney after its intravenous injection to normal mice, and then, almost disappeared from the organs until 60 min after injection. In the present study, we investigated the effect of cisplatin administration on the renal distribution of radioactivity in the initial phase after intravenous injection of [^3^H]hydromethidine to mice at 18 h after cisplatin administration (Fig. 2). The regional distribution of radioactivity in the kidney at 1 min after [^3^H]hydromethidine injection, which seemed to be dependent upon renal blood flow, showed no marked difference between the control and the cisplatin group as shown in Fig. 2. In addition, it has been reported that renal tissue damage after cisplatin administration progresses in a time-dependent manner [27, 32]. These data suggest that the renal blood flow was basically unchanged until 18 h after cisplatin administration. It has been also reported that DMTU had no effect on the renal blood flow in normal rats intravenously injected with DMTU at 500 mg/kg [33]. These data suggest that the uptake kinetics of [^3^H]hydromethidine is basically unchanged by treatment of cisplatin or DMTU. In the kidney of normal mice, radioactivity almost disappeared at 60 min after the injection of [^3^H]hydromethidine. In mice treated with cisplatin, significant accumulation of radioactivity at 60 min post-injection of the tracer was observed in the corticomedullary junction in a time-dependent manner after the cisplatin administration, as shown in Fig. 1. This finding suggests that ROS generation is kept at a normal level until 5 h after cisplatin administration, reaches an excess amount at 10–18 h and then triggers subsequent renal dysfunction and tissue damage. [^3^H]hydromethidine could detect excess renal ROS such as ^·^OH in cisplatin-induced nephrotoxicity in a similar manner to ROS overproduction by microinjection of SNP into the brain, which has been previously reported [22]. Furthermore, renal radioactivity accumulation in cisplatin-treated mice was attenuated by the DMTU pretreatment, indicating radioactivity accumulation after [^3^H]hydromethidine injection mainly reflects the amount of ^·^OH produced in the kidney. These findings suggest that [^3^H]hydromethidine is rapidly delivered into the kidney, converted to its highly polar form in the presence of ROS such as ^·^OH and intracellularly accumulated. We measured the MDA level, an index of lipid peroxidation, in the kidney obtained from the cisplatin-treated mice and confirmed that cisplatin administration increased the degree of lipid peroxidation, as shown in Fig. 5. Domitrović et al. recently reported the expression of 4-hydroxy-2-nonenal (4-HNE), one of the lipid peroxidation products, in the kidney of cisplatin-treated mice by immunohistochemical study [24]. In that report, highly immunopositive cells for 4-HNE were present in the corticomedullary junction of the kidneys from cisplatin-treated mice, whereas the kidneys of control mice were immunonegative for 4-HNE. Other lipid peroxidation markers, such as N^ε^-hexanoyl lysine and acrolein, also have been reported to be expressed in the corticomedullary area of the kidney from cisplatin-treated rats [32]. These reports suggest that ROS could exist and cause lipid peroxidation in the corticomedullary area of the kidney, where radioactivity accumulation after [^3^H]hydromethidine injection was observed. We also examined the effect of DMTU pretreatment against cisplatin-induced nephrotoxicity. The DMTU treatment before cisplatin administration suppressed the increase of serum creatinine and BUN and the severity of renal tissue damage in cisplatin-treated mice, as shown in Figs. 3 and 4. Furthermore, cisplatin-induced lipid peroxidation in the kidney was attenuated by the DMTU pretreatment, as shown in Fig. 5. Thus, ROS such as ^·^OH could be involved in cisplatin-induced nephrotoxicity.

Further studies including synthesis of ^11^C-labeled hydromethidine could enable PET imaging of ROS in living animals or humans and contribute to the understanding of the pathogenesis of cisplatin-induced nephrotoxicity.

## Conclusions

The present study showed that [^3^H]hydromethidine enables assessment of the regional ROS overproduction in mouse kidney treated with cisplatin as well as the effect of antioxidant treatment.
